# A fecundity cost of (walking) mobility in an insect

**DOI:** 10.1002/ece3.396

**Published:** 2012-10-04

**Authors:** Jörg Samietz, Günter Köhler

**Affiliations:** 1 Agroscope Changins-Wädenswil ACW, Zoology ResearchSchloss 1, Wädenswil, CH-8820, Switzerland; 2 Institute of Ecology, Friedrich-Schiller-UniversityJena, D-07743, Germany

**Keywords:** Cost of reproduction, dispersal, fecundity, fitness, mobility, movement ecology, Orthoptera, oviposition rate

## Abstract

Evolutionary theory predicts trade-offs between fecundity and mobility, but there is substantial lack of empirical evidence if and how basic mobility relates to fitness costs. In a field experiment, we investigated fecundity costs of mobility in a non-migratory, wing-monomorphic grasshopper, *Stenobothrus lineatus*, and at the same time tested for possible effects of reproductive state (egg-load) on the mobility. For 10 days, body weight and activity radius of 60 females were recorded daily and oviposition events were inferred from abrupt weight losses. We found a strong and significant relationship between the individual mobility and the time between egg pods laid (interpod period). Individual egg-laying was reduced by a rate of 0.36 eggs per day with each meter increase in mean daily activity radius. The trade-off was not biased by the size of the females, that is, constitution did not positively influence both offspring number and mobility. Egg-load had no significant influence on the individual distances travelled. We could demonstrate that mobility – as induced and selected for by foraging, thermoregulation, predator escape, shelter seeking, and reproduction – can be directly paid off by fecundity. This direct consequence of mobility on individual fitness was detected for the first time in a walking insect.

## Introduction

Evolutionary theory predicts that among the life-history parameters of an organism, fecundity should be optimized versus parameters allowing future reproductions by trade-offs to achieve optimum fitness (Stearns 1989; Kirkwood 2002). Besides body size and longevity, one important aspect of future investment in somatic maintenance is the potential to maintain mobility with its ecological and evolutionary consequences. However, current attempts to create an unifying paradigm of movement ecology (e.g., Dingle 1996; Clobert et al. 2001; Holyoak et al. 2008; Nathan et al. 2008) show that there is substantial lack of empirical evidence, especially from field populations in invertebrates, how regarding investments relate to each other.

First, it is hardly known if and how basic mobility transfers to fitness costs (Holyoak et al. 2008; Nathan et al. 2008). Second, the relationship between mobility and fecundity is little known outside the (relatively extreme) cases of migratory species (Rankin and Burchsted 1992; Dingle 1996) or species with dispersal-related wing-dimorphism (Denno 1994; Zera and Denno 1997). Finally, few attempts exist to separate two effects, that is, (1) the effect of mobility on the reproduction output as the basic trade-off, which could select for philopatry or non-dispersal behavior (e.g., Hanski et al. 2006; Gu et al. 2006; Saastamoinen 2007); and (2) the effect of reproductive state (egg-load) itself on the mobility (e.g., Olsson et al. 2000; Veasey et al. 2001; Plaut 2002).

**Figure 1:**
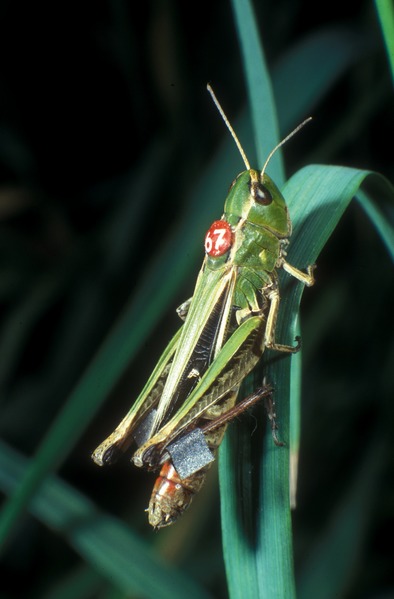
Female *Stenobothrus lineatus* with individually numbered marker on the pronotum and reflective tapes at the hind tibia (Photo: Frank Julich).

Here, we attempt to overcome these shortcomings in a field experiment in the non-migratory European grasshopper, *Stenobothrus lineatus* (Panzer) (Fig. 1). This species shows a philopatric behavior – it moves around its natal habitat and also disperses to neighboring suitable sites by walking. Both mobility and fecundity are important life-history components of *S. lineatus* (Samietz 1998; Bauer et al. 2005). The species is ideal for such an approach because in gomphocerine grasshoppers, fecundity is determined by the frequency by which egg pods are laid rather than by clutch size (Joern and Gaines 1990), both oviposition rate and mobility can be measured in the field (cf. Materials and Methods), and a possible interaction with body size can be detected if constitution would positively influence both mobility and fecundity.

## Materials and Methods

The field study was conducted during 10 consecutive days in a well-studied population in a southern-exposed Mesobrometum grassland (inclination 15–20°) (Leutratal near Jena/Germany 50.56°N, 11.35°E). In order to reduce effects of differences in microhabitat quality, we chose a homogenous study plot with an area of 1,300 m^2^_,_ which was covered with regularly distributed tufts of the grass *Bromus erectus*, used by *S. lineatus* as food and oviposition habitat (Samietz 1998). The isolation of the plot from neighboring grassland sites by dense shrub and tree borders amounted to about 55%. The management consisted of a yearly autumnal mowing with removal of biomass. The population size of *S. lineatus* at the study plot was estimated to about 200 individuals (Köhler 1999). The study period was chosen to represent the effective reproduction period of an average female in the field, which lays 3.7 egg pods and produces 23.5 eggs during 10.7 days after 8 days of maturation (Samietz 1998). During the morning of the first day (31 July), 60 matured females were caught within the study site and marked individually by means of colored bee markers glued on the pronota. In addition, we provided each individual with small pieces of self-adhesive reflective tape (Scotchlite7610, 3M, Neuss/Germany) on both hind tibiae in order to locate them at night by searching for the reflection signals with a headlamp (Fig. 1, cf. below). For exact localization of the grasshoppers at night, the entire plot was equipped with a square grid of 40-cm high poles (5 × 5 m distance of grid poles) marked with reflective tape around the top. The marked females were weighed and released in the center of the gridded area. Each subsequent evening, the area was carefully searched for marked females; position, mating status (copulations), and body weight (portable balance, ± 1 mg, Sartorius PT120-000V1, Göttingen/Germany) were recorded. Owing to the reflective tape, females were visible at night even at distances of about 50 m, thus the recapture probability of an individual during a night survey was above 90%.

**Figure 2:**
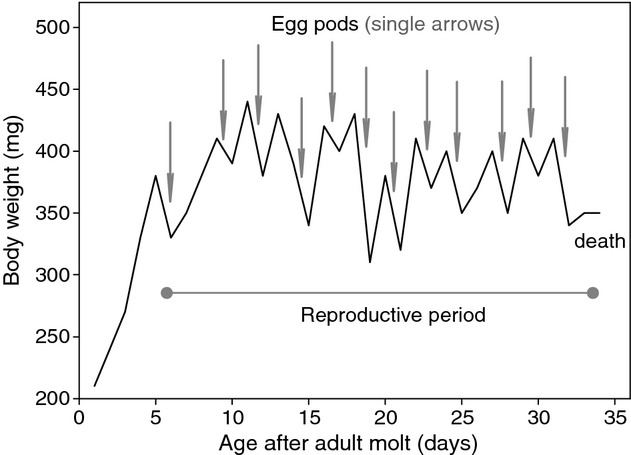
Oviposition sequence of a female *Stenobothrus lineatus* reared in the laboratory and checked for egg pods daily from adult molt to death.

Oviposition events in the field experiment were inferred from abrupt weight losses as validated in the laboratory before (Samietz 1998); an according oviposition sequence is exemplified for a female reared in the laboratory and checked for egg pods daily (Fig. 2). For those initial laboratory experiments, 40 pairs of *S. lineatus* were reared from hatching until their death at 30 ± 5°C and 70% relative humidity at long day (16L:8D) in single cages (15 × 15 × 35 cm) equipped with a 250-mL Erlenmeyer flasks containing dense bunches of daily fresh-cut *Dactylis glomerata* grass serving as food and oviposition habitat. Body weight of females was recorded daily in the morning (Sartorius PT120-000V1, Göttingen/Germany). At the same time, cages were checked for egg pods and the number of eggs was counted. The life-history parameters analyzed during laboratory experiments were lifetime fecundity (number of eggs), interpod periods (reciprocal oviposition rate per day), reproductive period (from first egg pod to death), longevity (from molting to death), and body size (mean body weight).

According to the laboratory experiment, oviposition had occurred after daily weight losses of at least 20 mg (cf. Fig. 2). This weight loss is about 5% of the body weight of mature females, but the precise figures depend on food intake and time of day and so cannot be used to infer exact clutch size in the field. The average weight loss related to oviposition events in the field experiment was 38 mg.

The duration of interpod periods in the field experiment was averaged for females that laid at least two egg pods and oviposition rates were calculated as their reciprocals. Mean activity radius, as the arithmetic average of the linear distances travelled per day, was calculated for subsequent analysis being a robust linear mobility parameter, relatively independent from the number of observation points (Samietz and Berger 1997).

The forward stepwise regression analysis and the linear regressions were carried out with SigmaPlot v. 11.0 (SysStat Software, Erkrath, Germany). All other statistical analyses were applied using XLStat Pro V2011.204 (Addinsoft, Andernach, Germany).

## Results

The initial laboratory experiments revealed that lifetime fecundity of the single females of *S. lineatus*, as the totals number of eggs laid, was best related to the reciprocal interpod period, that is, the oviposition rate per day (Table 1; Pearson's product moment correlation, *R* = 0.87, *P* < 0.0001), followed by reproductive period (*R* = 0.75, *P* < 0.0001). Among the other life-history parameters tested, body size (as mean body weight) was not correlated to fecundity, oviposition rate, reproductive period, and longevity (Table 1). Reproductive period and longevity were strongly interrelated (*R* = 0.93, *P* < 0.0001). A subsequent forward stepwise regression analysis confirmed that lifetime fecundity was predicted predominately by oviposition rate (coefficient = 114.5; *F* = 143.3, *P* < 0.0001), to some extent by reproductive period (coefficient = 1.20; *F* = 74.6, *P* < 0.0001), and not significantly by longevity (*F* = 0.56, *P* = 0.47) and body size (*F* = 0.27, *P* = 0.61).

**Table 1:** Correlation matrix of life-history parameters of *Stenobothrus lineatus* females in the laboratory (significant Pearson's coefficients in bold)

	Oviposition rate (egg pods per day)	Reproductive period (first egg pod to death)	Longevity (adult molt to death)	Body size (mean body weight)
Lifetime fecundity (number of eggs)	***R*** **=** **0.87**	***R*** **=** **0.75**	***R*** **=** **0.56**	*R* = 0.026
	*P* < 0.0001	*P* < 0.001	*P* < 0.05	*P* = 0.92
Oviposition rate		*R* = 0.38	*R* = 0.17	*R* = −0.17
		*P* = 0.11	*P* = 0.49	*P* = 0.49
Reproductive period			***R*** **=** **0.93**	*R* = 0.338
			*P* < 0.0001	*P* = 0.16
Longevity				*R* = 0.321
				*P* = 0.18

Oviposition events in the field experiment were identified in 50 *S. lineatus* females, of which 47 could be included in the mobility analysis owing to their sequence of recaptures. Their mean daily activity radius amounted to 4.25 ± 0.57 m (s.d.) with considerable variation from 1.60 to 9.24 m. Mean body weight of females was 400 ± 40 mg, varying from 300 to 470 mg over the study period.

We recorded 85 interpod periods with oviposition rates being on average 0.41 ± 0.14 egg pods per day. The arithmetic individual mean interpod period was 2.80 ± 1.06 days, varying from 1 to 6 days.

The mean interpod period was significantly influenced by the mean radius moved per day (full factorial ANCOVA, covariate, *F*_1,46_ = 36.50, *P* < 0.0001), but not by average body size (body weight quartiles, *F*_3,46_ = 0.174, *P* = 0.913). Females of different body sizes did not differ in their relationship between mobility and reproductive events (interaction, *F*_3,46_ = 1.20, *P* = 0.321).

**Figure 3:**
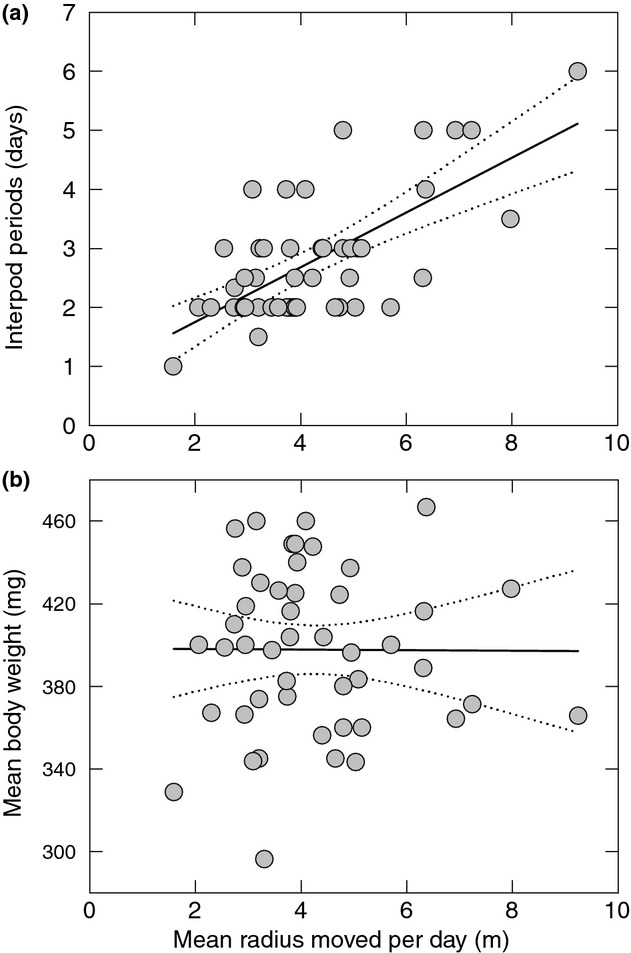
Linear regressions of (a) mean time between ovipositions (interpod periods) in female *Stenobothrus lineatus* grasshoppers (Pearson's *R* = 0.68, regression slope = 0.46, *t*_45_ = 6.1, *P* < 0.0001), and (b) mean body weight as measure for the females' size (Pearson's *R* = 0.006, regression slope = −0.00014, *t*_45_ = −0.038, *P* = 0.97) as functions of the average radius moved per day. Distributions did not differ from normality (Shapiro-Wilk, *P*_*a*_ = 0.267, *P*_*b*_ = 0.542). Dotted lines: 95% confidence interval of the regressions.

In the subsequent analyses, the individual mean interpod period showed a highly significant positive relation with the mean radius moved per day, that is, the greater the radius of daily movements, the longer the period between ovipositions (Fig. 3a). With every meter of increase in activity radius, the interpod period was 0.46 days longer, which corresponds to a reduction of 0.36 eggs per day with constant reproduction period (cf. above). The mean individual body weight was not related to the mobility (Fig. 3b).

Comparing the mean individual radii travelled during the 24 hours before oviposition (4.27 ± 2.31 m) with the mean radii 24 hours after oviposition (4.26 ± 2.44 m) revealed no differences (Paired *t*-test, mean diff.= 0.017 ± 1.51 m, *t*_46_ = 0.076, *P* = 0.94), that is, females that covered shorter distances before oviposition also moved less after oviposition and *vice versa*.

Comparing the radii of the females moved during the 48–24 hours before a copulation was observed during resight (4.21 ± 2.32 m) with the radii moved < 24 hours before copulation (5.01 ± 3.17 m) showed no differences (paired *t*-test, mean diff. = −0.80 ± 3.43 m, *t*_6_ = 0.617, *P* = 0.56). There were also no differences between the radii 24 hours after copulation (4.86 ± 2.86 m) and the radii 24 hours before copulation (paired *t*-test, mean diff. = 0.16 ± 4.70 m, *t*_6_ = 0.088, *P* = 0.93).

## Discussion

Investigating the effect of individual mobility on female fitness, we found in a non-migrating and wing-monomorphic grasshopper species that individual egg-laying reduces with increasing mean daily activity radius. This trade-off occurred during within-habitat or intra-population movement, obviously resulting as a product of day by day mobility, which is associated in female *S. lineatus* with foraging, thermoregulation, seeking shelter, escaping predators, mating, and egg-laying (Samietz 1998; Bauer et al. 2005). Thereby, neither egg-load nor mating status had an effect on mobility; the latter possibly due to the high proportion of males, ready to mate, that are actively searching for receptive females (Bauer et al. 2005). The trade-off was not biased by the size of the females, that is, constitution did not positively influence both offspring number and mobility. This direct consequence of mobility on individual fitness was detected for the first time in a walking insect.

The present findings coincide with the traditional explanation that internal energy resources are limited thus giving rise to such a trade-off (Harshman and Zera 2006). It is well known from migratory species, where any flight muscle production and flight event is costly and reducing the following reproductive output, for example, in locusts (Farrow 1990). In wing-dimorphic species, the macropterous form is significantly less fecund (and the reproduction is delayed) than the micropterous form, as known from grasshoppers, crickets, planthoppers, and others (Zera and Denno 1997). In *Cydia pomonella*, females of the sedentary genotype were larger, had a higher age-specific fecundity and lived longer than females of the mobile genotype (Gu et al. 2006). However, there are several notable exceptions both in migratory and in wing-dimorphic species, mainly in insects, providing some indication for physiological trade-offs between locomotor activity and reproduction. In the grasshopper *Melanoplus sanguinipes* and the moth *Spodoptera exempta,* such costs for long-duration flight or migration in gregaria morphs are avoided by a complex of physiological adaptations, hitherto not well understood (McAnelly and Rankin 1986; Rankin and Burchsted 1992; Zera and Harshman 2001). This holds true also for some wing-dimorphic species, where egg numbers of both forms do not differ within a population because long-winged females consumed additional food, for example, in *Gryllus firmus* (Mole and Zera 1994). In the butterfly *Melitaea cinxia*, in newly established populations (but not in old), mobile females had higher fecundities than less mobile females, explained by a physiological trade-off between high metabolic performance and reduced maximal life-span (Hanski et al. 2006; Saastamoinen 2007). However, our understanding of the mechanistic basis of the cost of reproduction is still limited, and several proximate mechanisms are still in discussion (Harshman and Zera 2006).

There are several examples with negative relations between body weight and mobility, where egg-load in gravid females reduces their mobility, as known for flight performance in birds (Lee et al. 1996; Veasey et al. 2001), locomotor activity in lizards (Sinervo et al. 1991; Shine et al. 1998; Olsson et al. 2000), snakes (Seigel et al. 1987), and fishes (Plaut 2002). Positive relations were detected in the wolf spider *Pardosa monticola*, where females transporting spiderlings were more mobile than other females (Bonte et al. 2007), and in the bug *Phyllomorpha laciniata*, where females lay eggs on male and female conspecifics, and this egg-load does not affect mobility and speed (Miettinen et al. 2006). In our experiment, we could exclude that egg-load of the females before oviposition hinders them in moving faster.

In this study, we were able to demonstrate that mobility within the habitat – as induced and selected for by foraging, thermoregulation, predator escape, shelter seeking, and reproduction – can have immediate fecundity costs, specifically by increasing the time intervals between ovipositions. This finding in grasshoppers represents a novel cost and implies that between-habitat movement, that is, colonization of neighboring habitats, by walking emigration is also likely to be costly. It provides a possible explanation for the widespread philopatric behavior in many invertebrates (e.g., Samietz and Berger 1997).

## References

[b1] Bauer S, Samietz J, Berger U (2005). Sexual harassment in heterogeneous landscapes can mediate population regulation in a grasshopper. Behav. Ecol.

[b2] Bonte D, Maelfait S, Van Belle J-P (2007). Maternal care and reproductive state-dependent mobility determine natal dispersal in a wolf spider. Anim. Behav.

[b3] Clobert J, Danchin E, Dhondt AA, Nichols JD (2001). Dispersal.

[b4] Denno RF (1994). The evolution of dispersal polymorphisms in insects – the influence of habitats, host plants and mates. Res. Popul. Ecol.

[b5] Dingle H (1996). Migration: the biology of life on the move.

[b6] Farrow RA, Chapman RF, Joern A (1990). Flight and migration in Acridoids. Biology of grasshoppers.

[b7] Gu H, Hughes J, Dorn S (2006). Trade-off between mobility and fitness in *Cydia pomonella* L. (Lepidoptera: Tortricidae). Ecol. Entomol.

[b8] Hanski I, Saastamoinen M, Ovaskainen O (2006). Dispersal-related life history trade-offs in a butterfly metapopulation. J. Anim. Ecol.

[b9] Harshman LG, Zera AJ (2006). The cost of reproduction: the devil in the details. Trends Ecol. Evol.

[b10] Holyoak M, Casagrandi R, Nathan R, Revilla E, Spiegel O (2008). Trends and missing parts in the study of movement ecology. Proc. Nat. Acad. Sci. USA.

[b11] Joern A, Gaines B, Chapman RF, Joern A (1990). Population dynamics and regulation in grasshoppers. Biology of grasshoppers.

[b12] Kirkwood TBL (2002). Evolution of ageing. Mech. Ageing Dev.

[b13] Köhler G (1999). Ökologische Grundlagen von Aussterbeprozessen. Fallstudien an Heuschrecken (Caelifera et Ensifera).

[b14] Lee SJ, Witter MS, Cuthill IC, Goldsmith AR (1996). Reduction in escape performance as a cost of reproduction in gravid starlings, *Sturnus vulgaris*. Proc. R. Soc. Lond. B.

[b15] McAnelly ML, Rankin MA (1986). Migration in the grasshopper *Melanoplus sanguinipes* (Fab.). II. Interactions between flight and reproduction. Biol. Bull.

[b16] Miettinen M, Kaitala A, Smith RL, Ordóñez RM (2006). Do egg carrying and protracted copulation affect mobility in the Golden Egg Bug?. J. Insect Behav.

[b17] Mole S, Zera AJ (1994). Differential resource consumption obviates a potential flight-fecundity trade-off in the sand cricket (*Gryllus firmus*. Funct. Ecol.

[b18] Nathan R, Getz WM, Revilla E, Holyoak M, Kadmon R, Saltz D (2008). A movement ecology paradigm for unifying organismal movement research. Proc. Nat. Acad. Sci. USA.

[b19] Olsson M, Shine R, Bak-Olsson E (2000). Locomotor impairment of gravid lizards: is the burden physical or physiological?. J. Evol. Biol.

[b20] Plaut I (2002). Does pregnancy affect swimming performance of female Mosquitofish, *Gambusia affinis*. Funct. Ecol.

[b21] Rankin MA, Burchsted JCA (1992). The cost of migration in insects. Annu. Rev. Entomol.

[b22] Saastamoinen M (2007). Mobility and lifetime fecundity in new versus old populations of the Glanville fritillary butterfly. Oecologia.

[b23] Samietz J (1998). Populationsgefährdungsanalyse an einer Heuschreckenart – Methoden, empirische Grundlagen und Modellbildung bei Stenobothrus lineatus (Panzer).

[b24] Samietz J, Berger U (1997). Evaluation of movement parameters in insects – bias and robustness with regard to resight numbers. Oecologia.

[b25] Seigel RA, Huggins MM, Ford NB (1987). Reduction in locomotor ability as a cost of reproduction in gravid snakes. Oecologia.

[b26] Shine R, Keogh S, Doughty P, Giragossyan H (1998). Costs of reproduction and the evolution of sexual dimorphism in a ‘Flying lizard’ *Draco melanopogon* (Agamidae). J. Zool.

[b27] Sinervo B, Hedges R, Adolph SC (1991). Decreased sprint speed as a cost of reproduction in the lizard *Sceloporus occidentalis* - Variation among populations. J. Exp. Biol.

[b28] Stearns SC (1989). Trade-offs in life-history evolution. Funct. Ecol.

[b29] Veasey JS, Houston DC, Metcalfe NB (2001). A hidden cost of reproduction: the trade-off between clutch size and escape take-off speed in female zebra finches. J. Anim. Ecol.

[b30] Zera AJ, Denno RF (1997). Physiology and ecology of dispersal polymorphism in insects. Annu. Rev. Entomol.

[b31] Zera AJ, Harshman LG (2001). Physiology of life history trade-offs in animals. Annu. Rev. Ecol. Syst.

